# Cancer Research in the “Chemical Biology” Section of the Journal *Molecules*

**DOI:** 10.3390/molecules25225275

**Published:** 2020-11-12

**Authors:** Timothy W. Corson

**Affiliations:** Departments of Ophthalmology, Biochemistry and Molecular Biology, and Pharmacology and Toxicology, and the Melvin and Bren Simon Comprehensive Cancer Center, Indiana University School of Medicine, Indianapolis, IN 46202, USA; tcorson@iu.edu; Tel.: +1-317-274-3305

The Chemical Biology Section of *Molecules*, like the discipline it represents, is diverse, dynamic, and growing rapidly. As *Molecules* celebrates its 25th anniversary this year, the Chemical Biology Section has published nearly 500 articles, overseen by an editorial board of 66 scientists representing a variety of subdisciplines and working in 20 different countries. A valuable feature of the journal is the special issue program; these special issues offer enhanced visibility and thematic groupings of articles. As of this writing, there are 43 Chemical Biology Section special issues currently accepting submissions, grouped around various techniques, pathways, classes of molecules, and disease areas.

Amongst these disease areas is cancer research, a domain that has had a long and successful relationship with chemical biology. Natural product mechanistic studies led to anticancer drugs like taxanes [1] and proteasome inhibitors [2]. Structural studies led to molecularly targeted therapies, as originated by imatinib for chronic myelogenous leukemia [3]. Such therapies are now legion. More recently, targeted protein degradation via PROTACs and related technology has transitioned from a research tool to a cancer therapeutic modality [4]. Moreover, chemical biology methods have led to countless other research tools used in cancer laboratories, from phenotypic small molecule screening methods [5], to chemical probes such as bromodomain inhibitor JQ1 [6], to workhorse assays such as EdU S-phase labeling [7], to imaging tools for use at the molecular to organismal levels. Building on this rich history, several articles from the last two years highlight the diversity and scope of cancer research published in the Chemical Biology Section of *Molecules*, both primary literature and reviews.

Triple-negative breast cancer (TNBC) is a hard-to-treat breast cancer subtype. Chen et al. [8] presented a combination therapy study of TNBC, pairing a standard of care cytotoxic chemotherapeutic, doxorubicin, with the natural product alkaloid piperlongumine. Piperlongumine synergized with doxorubicin in cultured cells and in a xenograft model, promoting apoptosis and reducing JAK-STAT signaling, revealing a potential mechanism of action.

In another combination study, Spengler et al. [9] explored the effects of organoselenium compounds on modulating multidrug-resistant murine T lymphoma cell sensitivity to chemotherapeutics. They developed a series of novel selenocompounds that can inhibit the ABCB1 multidrug efflux pump and showed that some of these synergized with chemotherapies, notably vincristine. These findings raise exciting questions for exploration in further mechanistic and animal efficacy studies.

Further focusing on transport proteins, Juraszek and Nałęcz [10] reviewed the expression regulation, subcellular localization, protein-protein interactions, and transport activity of the OCTN2 principal carnitine transporter. Carnitine is a valuable alternative energy source for β-oxidation in cancer cells. The authors highlighted the overexpression of OCTN2 in some cancers, and how this metabolic adaptation could be used against cancer cells, since OCTN2 allows uptake of chemotherapeutics and other drugs.

Effective modeling of cancer in vitro remains a challenge. Balmaña et al. [11] developed methods for creating 3D spheroids of four gastric cancer cell lines and explored how 3D growth affects glycosylation of the cells, a key phenotype relevant to tumor progression. Automated image analysis allowed assessment of spheroid parameters and lectin staining provided glycosylation profiles. Glycosylation inhibitors blocked growth of the spheroids, and mucin expression paralleled that seen in humans. This work both provided new methods and explored the glycobiology of gastric cancer.

An excellent example of a chemical biology tool with therapeutic potential is cell penetrating peptides (CPPs). Habault and Poyet [12] reviewed CPPs with an eye to cancer therapy. They highlighted the various classes of CPPs, examples of CPPs used in clinical development, and how CPPs can be applied to deliver chemotherapeutics, nucleic acids such as siRNAs, and therapeutic peptides or proteins into cells. Finally, they discussed optimization of CPPs, in particular promising strategies for tumor homing or cancer cell-specific activation.

However, not all chemical biology work is done at the lab bench. Wang et al. [13] brought a bioinformatic approach to the burgeoning circular RNA (circRNA) field. They described CSCRSites, a deep learning method to identify cancer-specific RNA binding protein (RBP) sites on circRNAs. RBPs appear to be essential for circRNA function, and CSCRSites performs better at predicting these binding sites than other tools, providing a valuable hypothesis-generating method for studying these intriguing molecules in cancer.

Seeking new compounds with desired biological activities is one of the fundamental goals of chemical biology. Merarchi et al. [14] reviewed natural products with activity as histone deacetylase (HDAC) inhibitors. HDAC inhibitors are of great interest in cancer therapy, given HDAC dysregulation in tumors. Adding to approved HDAC inhibitor drugs such as vorinostat, the authors highlighted several natural compounds with HDAC inhibitory activity, both pleiotropic and specific.

Of course, synthetic chemistry is a mainstay of chemical biology as well. Arumugam et al. [15] demonstrated this with an elegant multicomponent domino synthesis of a series of dispirooxindolopyrrolidines. Use of an ionic liquid, 1-butyl-3-methylimidazolium bromide, optimized the process and avoided classical solvents. Some of the resulting compounds induced apoptosis of U-937 lymphoma cells and docked to the proapoptotic protein Bcl-X_L_, suggesting potential cancer relevance.

As these varied articles attest, *Molecules* provides a valuable forum for presenting important chemical biology research and critical review articles of wide interest. Moreover, the journal’s impact factor (3.3 in 2019) compares favorably with that of other, similar chemical biology primary research-focused journals such as *ChemBioChem* (2.6), *Chemical Biology & Drug Design* (2.5), and *ACS Chemical Biology* (4.4). But impact factor should not be the only influence in choosing a journal in which to publish. Another consideration is accessibility: unlike other established journals in the field, *Molecules* provides gold open access for all articles. This ensures that published work is immediately available to interested readers worldwide; the article processing charge of 2000 CHF (about $USD2200) is very competitive. This accessibility translated to 11,192,808 downloads/views of full text manuscripts in 2019, more than 1000 per article published. Finally, *Molecules* and the Editorial Board members are committed to rapid turnaround, while maintaining rigorous and fair peer review; the average time from submission to publication is a mere 32 days.

From bioinformatic tools to glycobiology, drug synergy to synthesis, and chemical probe discovery to metabolism, chemical biology is an expanding, amorphous field. The Chemical Biology Section of *Molecules* is well poised to continue to host exciting advances in the field.
